# Acetylation at lysine 346 controls the transforming activity of the HTLV-1 Tax oncoprotein in the Rat-1 fibroblast model

**DOI:** 10.1186/1742-4690-10-75

**Published:** 2013-07-23

**Authors:** Julie Lodewick, Carla Sampaio, Mathieu Boxus, Anne-Sophie Rinaldi, Katia Coulonval, Luc Willems, Pierre P Roger, Françoise Bex

**Affiliations:** 1 Institute for Microbiological Research J-M Wiame (IRMW), Laboratory of Microbiology, Université Libre de Bruxelles, 1, Avenue E. Gryson, Brussels, Belgium; 2 National Fund for Scientific Research, Molecular Biology, GIGA and Gembloux Agro-Bio Tech, Université de Liège, Liège, Belgium; 3 Institute of Interdisciplinary Research (IRIBHM), Université Libre de Bruxelles, Campus Erasme, Brussels, Belgium

**Keywords:** HTLV-1, Tax, Acetylation, CDK4, Transformation, Leukemia

## Abstract

**Background:**

Transformation by the Tax oncoprotein of the human T cell leukemia virus type 1 (HTLV-1) is governed by actions on cellular regulatory signals, including modulation of specific cellular gene expression via activation of signaling pathways, acceleration of cell cycle progression via stimulation of cyclin-dependent kinase activity leading to retinoblastoma protein (pRb) hyperphosphorylation and perturbation of survival signals. These actions control early steps in T cell transformation and development of Adult T cell leukemia (ATL), an aggressive malignancy of HTLV-1 infected T lymphocytes. Post-translational modifications of Tax by phosphorylation, ubiquitination, sumoylation and acetylation have been implicated in Tax-mediated activation of the NF-κB pathway, a key function associated with Tax transforming potential.

**Results:**

In this study, we demonstrate that acetylation at lysine K_346_ in the carboxy-terminal domain of Tax is modulated in the Tax nuclear bodies by the acetyltransferase p300 and the deacetylases HDAC5/7 and controls phosphorylation of the tumor suppressor pRb by Tax-cyclin D3-CDK4-p21^CIP^ complexes. This property correlates with the inability of the acetylation deficient K_346_R mutant, but not the acetylation mimetic K_346_Q mutant, to promote anchorage-independent growth of Rat-1 fibroblasts. By contrast, acetylation at lysine K_346_ had no effects on the ability of Tax carboxy-terminal PDZ-binding domain to interact with the tumor suppressor hDLG.

**Conclusions:**

The identification of the acetyltransferase p300 and the deacetylase HDAC7 as enzymes modulating Tax acetylation points to new therapeutic targets for the treatment of HTLV-1 infected patients at risk of developing ATL.

## Background

The oncogenic retrovirus human T cell leukemia virus type 1 (HTLV-1) causes both Adult T cell leukemia (ATL), a fatal malignancy that occurs in 2 to 4% of infected carriers after decades of asymptomatic infection, and a neurodegenerative disease called HTLV-1 associated myelopathy/tropical spastic paraparesis (HAM/TSP) [1-3]. Early steps in the transformation of CD4^+^ T lymphocytes by HTLV-1 have been associated with the oncogenic properties of its protein Tax.

Four central activities have been linked to Tax transforming potential. First, Tax activates cellular signaling pathways, including the canonical [4-9] and non-canonical NF-κB [10-13], the SRF [14-16] and the AP1 [17,18] pathways. This activity determines the expression of cellular genes involved in proliferation and differentiation of T lymphocytes. Second, Tax compromises genome stability by modulating the timing of replication origin activation and induction of reactive oxygen species (ROS) leading to generation of double strand breaks (DSB) [19,20]. In addition, Tax restricts appropriate response to DNA damages and suppresses apoptotic signals by inactivating the tumor suppressor p53 [21-25]. Third, Tax activates the pRb kinase activity of CDK4/6-cyclin D3-p21^CIP^ complexes, resulting in pRb hyperphosphorylation and proteasomal degradation. This activity leads to the release of active E2F transcription factor and acceleration of cell cycle progression at the G1 to S restriction point [26-30]. Fourth, Tax interacts with PDZ domain-containing proteins, including the tumor suppressors human homolog of Drosophila discs large protein (hDLG), Scribbe (hScrib) and MAGI-1, via its carboxy (C)-terminal PDZ binding motif (PBM) E_350_TEV_353._ These interactions result in perturbation of hDLG, hScrib and MAGI-1 functions in cell growth control [31-36]. The contribution of each of these oncogenic activities to Tax-mediated transforming phenotype and establishment of the leukemogenic process remains debatable. In addition, the observations that Tax expression is frequently turned off in leukemic T lymphocytes from ATL patients, and that the HTLV-1 bZIP factor (HBZ) promotes proliferation of ATL cells through its mRNA form, clouds the role of Tax in the maintenance of the transformed state [37].

Post-translational modifications of Tax, in the form of phosphorylation, poly-ubiquitination, and poly-sumoylation control both Tax intracellular localization as well as Tax-mediated activation of the NF-κB pathway [38-42]. This involves cooperation between phosphorylation dependent poly-ubiquitinated cytoplasmic forms of Tax and poly-sumoylated nuclear forms, resulting in the assembly of transcriptionally active Tax nuclear bodies (NBs) containing the RelA subunit of NF-κB and the transcriptional coactivator p300 [43]. The contribution of both ubiquitination and sumoylation in Tax-mediated activation of the NF-κB pathway has been recently challenged [44] and further discussed [45,46].

Our group has previously reported that the lysine residue at amino acid position 346 (previously designated K10) in the C-terminal domain of Tax is the target for acetylation by the acetyltransferase activity of p300. Acetylation of Tax favors transcription initiated at a chromatin-integrated, but not on a transiently transfected, NF-κB controlled promoter [47].

The acetylation targeted lysine K_346_ is part of the 20 C-terminal amino acids of Tax, which have been directly implicated in Tax transforming activity. This region includes the PBM motif involved in Tax interaction with PDZ domain-containing proteins [33,34] and a domain involved in genomic instability as evidenced by micronuclei formation [48,49]. In this work, we demonstrate that K_346_ acetylation controls the ability of Tax to transform Rat-1 fibroblasts in the well-established model of colony formation in soft agar. Transformation of Rat-1 fibroblasts by acetylated Tax correlates with stimulation of the kinase activity of CDK4-cyclin D3-p21^CIP^ complex, leading to pRb phosphorylation.

## Results

### Two acetylated forms of Tax are detected in various cell lines expressing Tax, including HTLV-1 infected T lymphocytes

**Figure 1:**
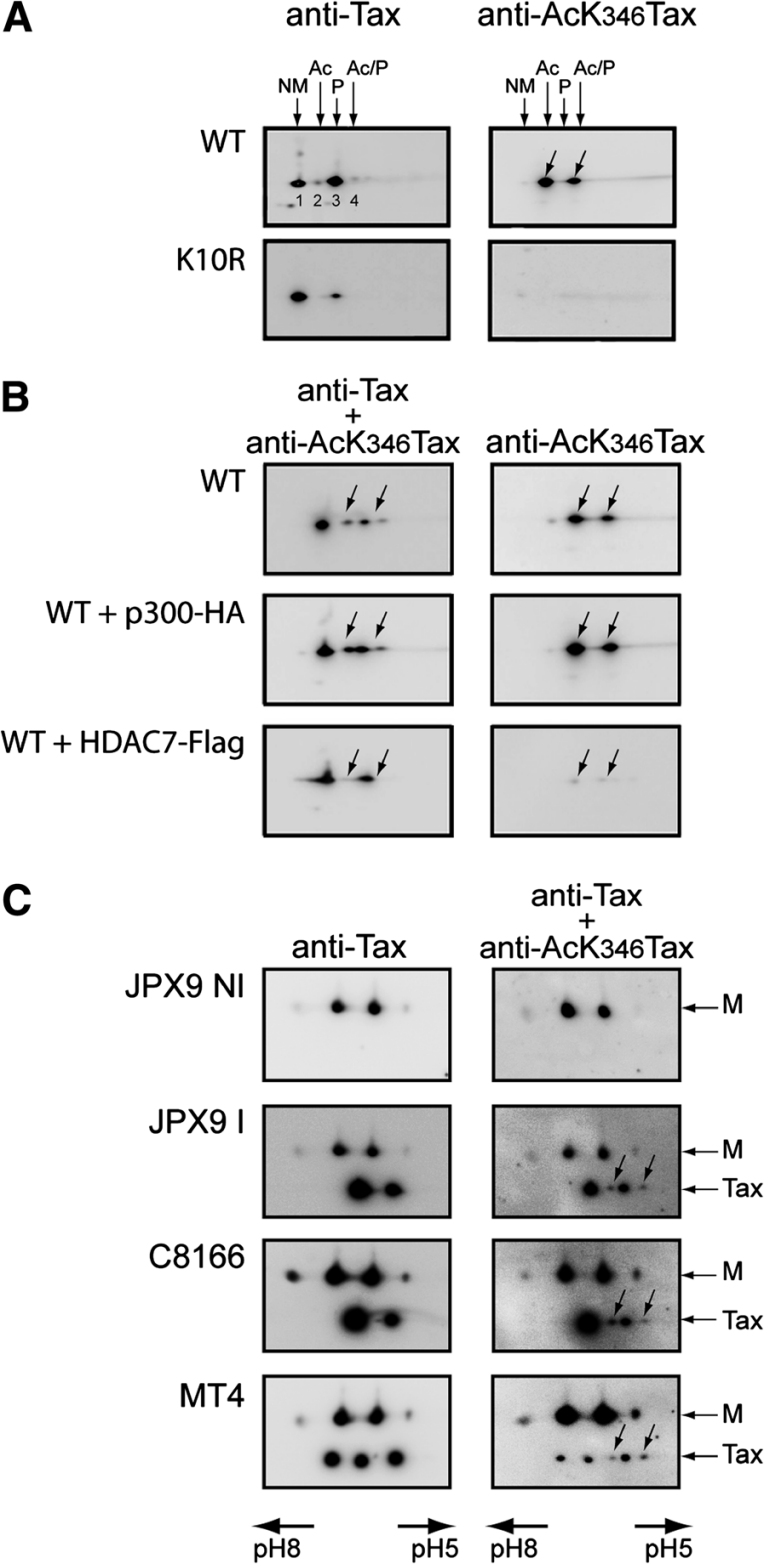
**Two acetylated forms of Tax are detected in various cell lines expressing Tax, including HTLV-1 infected T lymphocytes.** Extracts from 293T cells expressing WT Tax or the K_346_R mutant **(A)**, WT Tax and either p300-HA or HDAC7-Flag **(B)**, or JPX9 T lymphocytes either induced for Tax expression with CdCl_2_ (I) or not induced (NI) and HTLV-1 infected T lymphocyte cell lines C8166 and MT4 **(C)**, were analyzed by two-dimensional gel electrophoresis followed by Western Blotting with anti-Tax and anti-AcK_346_Tax antibodies. M, internal pH marker; NM, non-modified; P, phosphorylated; Ac, acetylated; P/Ac, phosphorylated and acetylated. Acetylated forms are highlighted by arrows.

Acetylation of a lysine residue modifies the isoelectric pH of proteins. Consequently, we used two-dimensional gel electrophoresis followed by Western Blotting (2D-Western) to identify the acetylated forms of Tax in lysates of 293T cells (Figure 1A and 1B) or T lymphocyte cell lines expressing Tax (Figure 1C). A monoclonal antibody (mAb) directed against Tax (anti-Tax) and a newly developed rabbit polyclonal antibody directed against the form of Tax acetylated at K_346_ (anti-AcK_346_Tax) were used to detect the various forms of Tax. Detection using the anti-Tax antibody revealed four species of molecular mass 40 kDa in cells expressing wild type (WT) Tax (annotated 1, 2, 3 and 4). Among them, the two barely detected forms (2 and 4) were detected in WT Tax but not in K_346_R mutant expressing cells. When the same samples were analyzed by 2D-Western with the anti-AcK_346_Tax antibody, two species of Tax were detected in WT Tax expressing cells, but no form of Tax was detected in cells expressing mutant K_346_R. The alignment with an internal isoelectric point marker (not included in the area of gels presented in Figure 1A) indicated that the two acetylated species detected by the anti-AcK_346_Tax antibody in WT Tax expressing cells comigrated with species 2 and 4 detected by the anti-Tax antibody.

To further characterize the acetylated forms of Tax, WT Tax was coexpressed with either the acetyltransferase p300 or the class IIa deacetylase HDAC7 and analyzed by 2D-Western (Figure 1B). Coexpression of p300 with WT Tax markedly increased the intensity of forms 2 and 4, whereas coexpression of HDAC7 resulted in the disappearance of these two acetylated forms. Coexpression of HDAC5, another class IIa deacetylase also led to reduced acetylation of Tax, contrary to coexpression of the class IIb deacetylase HDAC6 (data not shown). Furthermore, treatment of Tax expressing 293T cells with the deacetylase inhibitor Trichostatin A (TSA) resulted in a two fold increase of Tax acetylation (Additional file 1).

It is worthwhile noting that the two more acidic species of Tax detected by 2D-Western using the anti-Tax antibody (species 3 and 4) are phosphorylated, as indicated by metabolic labeling with ^32^P orthophosphate (Additional file 2). Thus, separation of the modified forms of Tax by 2D-Western revealed four different 40 kDa species including a non-modified form (NM, form 1), an acetylated form (Ac, form 2), a phosphorylated form (P, form 3) and an acetylated and phosphorylated form (Ac/P, form 4), with estimated percentages of NM 46%, Ac 3%, P 50% and Ac/P 1% (Additional file 2).

We next wanted to determine whether the two acetylated forms of Tax were also present in T lymphocyte cell lines expressing Tax. JPX-9 cells, that express Tax after induction with CdCl_2_, or HTLV-1 infected T lymphocyte cell lines, C8166 and MT4 were analyzed by 2D-Western (Figure 1C). Each of these cell lines contained the Ac and Ac/P forms of Tax, in addition to the NM and P forms. The MT4 cell lines also expressed additional forms that did not comigrate with the NM, Ac, P and Ac/P forms identified above, possibly due to the presence in this cell line of multiple proviruses that express variant Tax proteins.

### Acetylation of Tax is modulated by p300 and HDAC7 in the Tax nuclear bodies

**Figure 2:**
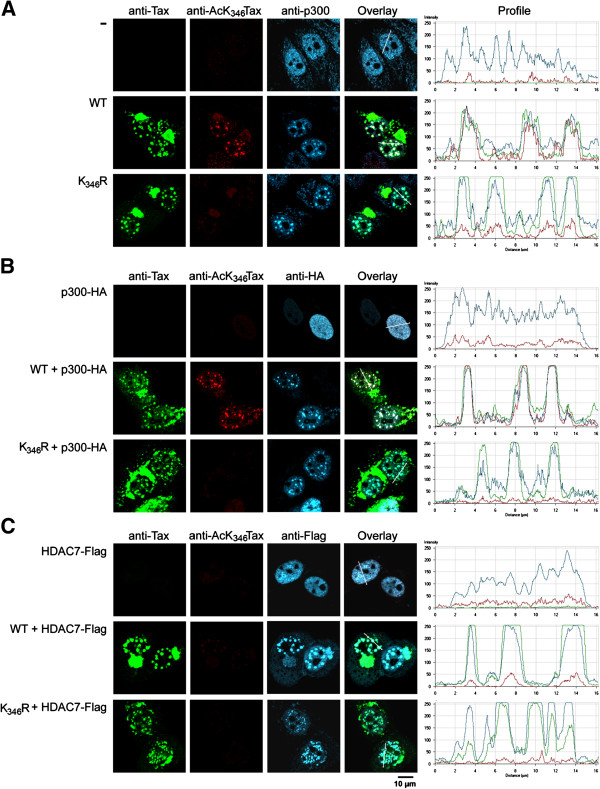
**Colocalization of Tax with p300 and HDAC7 in the Tax nuclear bodies modulates Tax acetylation status.** HeLa cells were transfected with vectors expressing WT Tax or the K_346_R mutant alone **(A)** or with a vector expressing either p300-HA **(B)** or HDAC7-Flag **(C)**. Cells were analyzed by triple immunofluorescence staining and confocal microscopy with the anti-Tax IgG2a mAb, the anti-AcK_346_Tax rabbit polyclonal antibody and either the anti-p300 IgG1 mAb **(A)**, the anti-HA IgG1 mAb for the detection of overexpressed p300-HA **(B)** or the anti-Flag IgG1 mAb for the detection of HDAC7-Flag **(C)**. The intensity profiles of the fluorescence staining along lines crossing the nuclei are shown.

Our previous studies indicated that both WT Tax and the acetylation deficient K_346_R mutant had similar intracellular distribution and colocalized with the acetyltransferase p300 in Tax NBs. In addition, cellular fractionation indicated that the acetylated form of Tax was predominantly localized in the nucleus, suggesting that the acetylated form of Tax could be present in the Tax NBs [47]. To directly test this possibility, HeLa cells expressing WT Tax or the K_346_R mutant were analyzed by immunofluorescence staining and confocal microscopy with the anti-AcK_346_Tax antibody (Figure 2A). Since overexpression of p300 increased the amount of the acetylated forms of Tax, whereas overexpression of HDAC7 resulted in the disappearance of these forms (see above), we included cells that coexpressed WT Tax or the K_346_R mutant with either p300-HA (Figure 2B) or HDAC7-Flag (Figure 2C). The intensity profiles of the immunofluorescence staining along lines drawn across the nuclei are shown.

Endogenous p300 had a speckled distribution in the nucleoplasm of cells in the absence of Tax expression. Expression of Tax confirmed the formation of Tax NBs containing endogenous p300 in WT Tax and mutant K_346_R expressing cells [47,50]. Interestingly, anti-AcK_346_Tax antibody detected the acetylated form of Tax in the Tax NBs formed by WT Tax, whereas no staining was observed in the Tax NBs assembled by mutant K_346_R (Figure 2A). Cytoplasmic Tax molecules that accumulated at the boundary of the nuclear envelope were detected only by anti-Tax and not by anti-AcK_346_Tax antibody in both wild type and mutant K_346_R expressing cells.

Overexpressed p300-HA concentrated in the Tax NBs and markedly increased the intensity of the anti-AcK_346_Tax fluorescence staining in the Tax NBs formed by WT Tax as shown by the intensity profiles in Figure 2A (WT) and Figure 2B (WT + p300-HA). Staining intensity was at background level in mutant K_346_R expressing cells. Overexpressed HDAC7-Flag (or HDAC5-Flag, data not shown) had a rather diffuse distribution in the nucleoplasm in cells that did not express Tax and concentrated in the Tax-NBs formed by WT and mutant K_346_R Tax (Figure 2C). Concentration of HDAC7-Flag in the Tax NBs resulted in a marked reduction of the anti-AcK_346_Tax fluorescence staining in the Tax NBs. The same results were obtained when HDAC5-Flag was expressed instead of HDAC7-Flag, whereas expression of the cytoplasmic deacetylase HDAC6 did not colocalize with Tax in the Tax NBs and had no effect on the level of acetylated Tax detected in Tax NBs (data not shown). These results demonstrated that the acetylated forms of Tax were present in the Tax NB. They also supported the idea that the acetylation status of Tax was modulated by p300 and HDAC7/5 in the Tax NBs. Furthermore, WT Tax and the acetylation deficient mutant K_346_R were similarly distributed in cytoplasmic hot spots and in nuclear bodies and overexpression of p300 or HDAC7, which have opposite effects on Tax acetylation status (see Figure 1), did not affect Tax intracellular localization. From these observations, we concluded that Tax acetylation status does not control Tax intracellular localization.

### Acetylation controls Tax-mediated transformation of Rat-1 cells

Lysine K_346_ is part of the C-terminal domain of Tax involved in Tax transforming activity. We consequently tested whether acetylation controlled Tax ability to induce anchorage-independent growth of Rat-1 fibroblasts, a well-established method to determine Tax transforming potential. First, based on the previous observation that a glutamine residue mimics acetylated lysines [51], we constructed a Tax mutant in which lysine K_346_ was replaced by a glutamine residue (mutant K_346_Q). Mutant K_346_Q had a distribution very similar to WT Tax or mutant K_346_R, with concentration in cytoplasmic structures associated with the Golgi and colocalization with p300 in nuclear bodies. As expected, this mutant was not detected by the anti-AcK_346_Tax antibody (data not shown). Then, Rat-1 cells were stably transduced with either lentiviral control vector (Rat/LV) or lentiviral vectors expressing WT Tax (Rat/LV-WT), mutant K_346_R (Rat/LV- K_346_R) or mutant K_346_Q (Rat/LV-K_346_Q).

**Figure 3:**
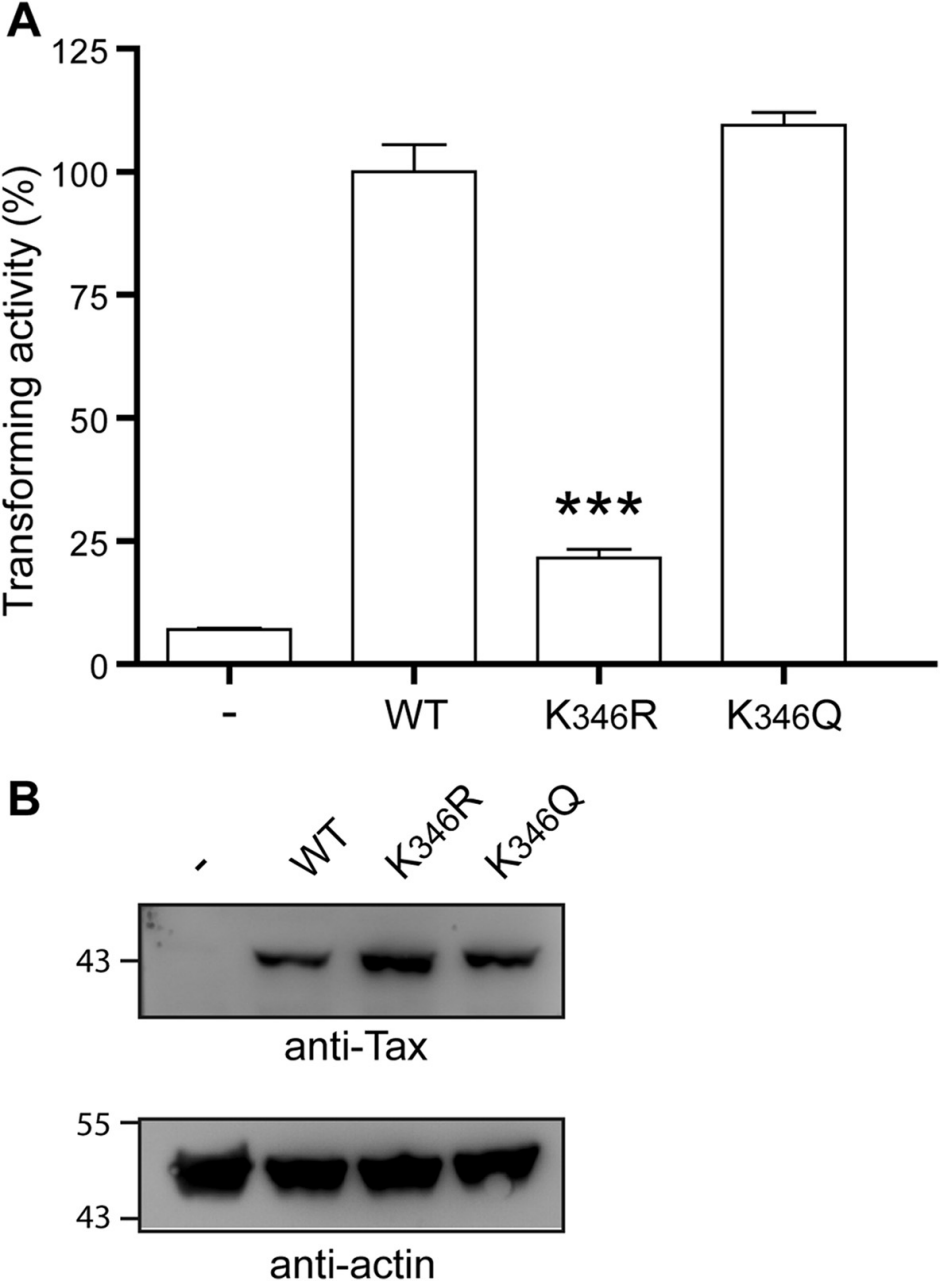
**Acetylation controls Tax-mediated transformation of Rat-1 cells. (A)** Rat-1 cells were stably transduced with lentiviral vectors expressing WT Tax or the K_346_R or K_346_Q mutants. Cells were plated in soft agar and colonies were counted 6 weeks later. The transforming activity expressed as the percentages of colonies formed relatively to WT Tax are the average of 3 independent experiments. ***: p < 0.05. **(B)** Before plating, aliquots of the Rat-1 cells transduced with the lentiviral vectors were lysed and tested for expression of Tax by Western Blot with anti-Tax and anti-actin antibodies.

FACS analysis was used to determine that about 75% of the transduced Rat-1 cells expressed WT Tax or K_346_R or K_346_Q after 7 days of culture in the presence of 10 μg/ml of blasticidin (data not shown). The cells were plated in soft agar and the number of colonies formed after 6 weeks was estimated (Figure 3A). Aliquots of the transduced Rat-1 cells were also tested for expression of WT or mutant Tax (Figure 3B). The percentage of anchorage-independent colonies formed by Rat/LV-K_346_R was only 20% as compared to Rat/LV-WT, whereas Rat/LV-K_346_Q formed anchorage-independent colonies at the same rate as Rat/LV-WT Tax. Quantitation of WT or mutant Tax expressed in the transduced Rat-1 cells indicated that the reduced transformation rate of Rat/ LV-K_346_R did not result from reduced expression of this mutant. In addition, the Tax nucleotide sequence of three transformed Rat/LV-WT, Rat/LV-K_346_R and Rat/LV-K_346_Q clones was determined to assure the correct WT or mutated sequence at residue 346. We concluded that acetylation controls Tax transforming activity in Rat-1 fibroblasts.

### Transformation of Rat-1 cells does not depend on Tax-mediated activation of the NF-κB pathway

**Figure 4:**
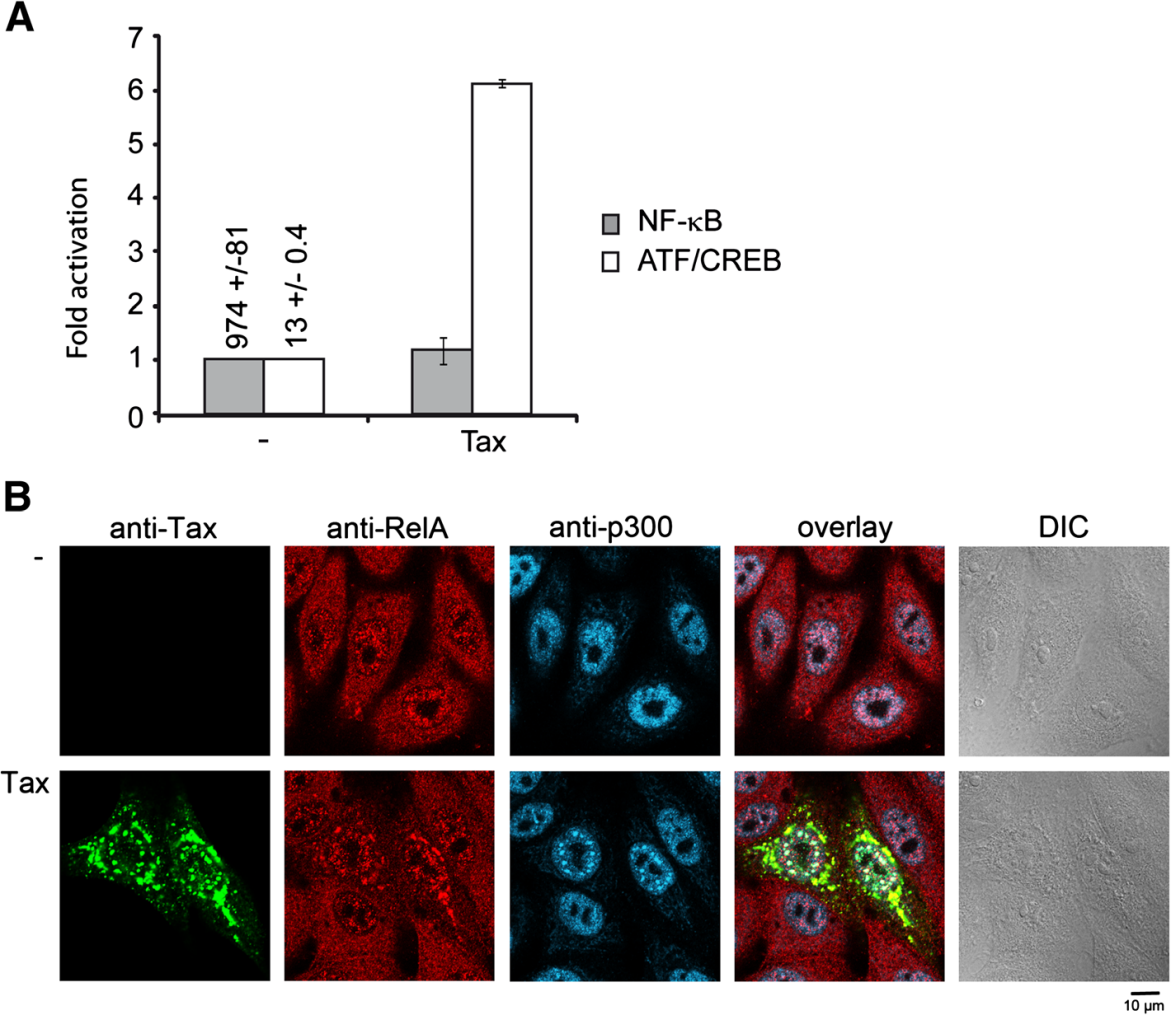
**Activation of the NF-κB and ATF/CREB pathways in Rat-1 cells expressing wild type Tax. (A)** Rat-1 cells transducted with either the control lentiviral vector (−) or with the lentiviral vector expressing wild type Tax (Tax) were transfected with 0.5 μg of κB-luc (grey bars) or 0.5 μg HTLV-1 LTR-luc (white bars) reporter constructs and 0.5 μg of pRL-TK-luc. The results are presented as fold increase of luciferase activities in Rat-1 cells expressing Tax relatively to control Rat-1 cells. The values are averages of three independent experiments, with standard deviations. The numbers above the bars represent basal luciferase activities in control Rat-1 cells. **(B)** Control Rat-1 cells (−) or Rat-1 cells expressing Tax (Tax) were analyzed by triple immunofluorescence staining with anti-Tax IgG2a mAb, anti-RelA rabbit polyclonal antibody and anti-p300 IgG1 mAb followed by confocal microscopy.

Activation of the NF-κB pathway is one of the Tax functions critical for cellular transformation [52,53]. Therefore, we tested the ability of Tax to activate the NF-κB pathway in the Rat/LV-WT cells by transfection of a κB-luc reporter construct. Tax-mediated activation of the HTLV-1 promoter via the ATF/CREB pathway was also tested as a control for Tax transcriptional activity by transfection of the HTLV-1 LTR-luc reporter construct (Figure 4A). WT Tax expressed in the Rat-1 transduced cells was unable to increase basal NF-κB activity although it increased activation of transcription initiated at the HTLV-1 LTR by a factor of 6. Interestingly, comparison of the luciferase activities resulting from activation of the NF-κB and ATF/CREB pathways in control Rat-1 cells (974 versus 13 light units) strongly suggested that the NF-κB pathway was constitutively activated in Rat-1 cells, independently of Tax expression. To test this possibility, we checked whether the RelA subunit of NF-κB was present in the nuclei of Rat-1 cells that did not express Tax, by using immunofluorescence staining and confocal microscopy. The images presented in Figure 4B clearly indicated the RelA was present in the nuclei of Rat-1 cells. Expression of Tax in these cells resulted in the colocalization of RelA and p300 in the Tax NBs, like previously observed [47], but did not alter the level of RelA already present in the nuclei. Thus, transformation of Rat-1 cells appears to be independent of Tax-mediated NF-κB activation, but might depend on constitutive activation of the NF-κB pathway in these cells. This observation strongly suggests that the previously described 50% reduction in activation of a NF-κB-controlled promoter integrated in the chromatin by mutant K_346_R [47] cannot justify the loss of transforming activity of this mutant.

### Acetylation does not control the interaction of Tax with the PDZ domain-containing proteins, hDLG

**Figure 5:**
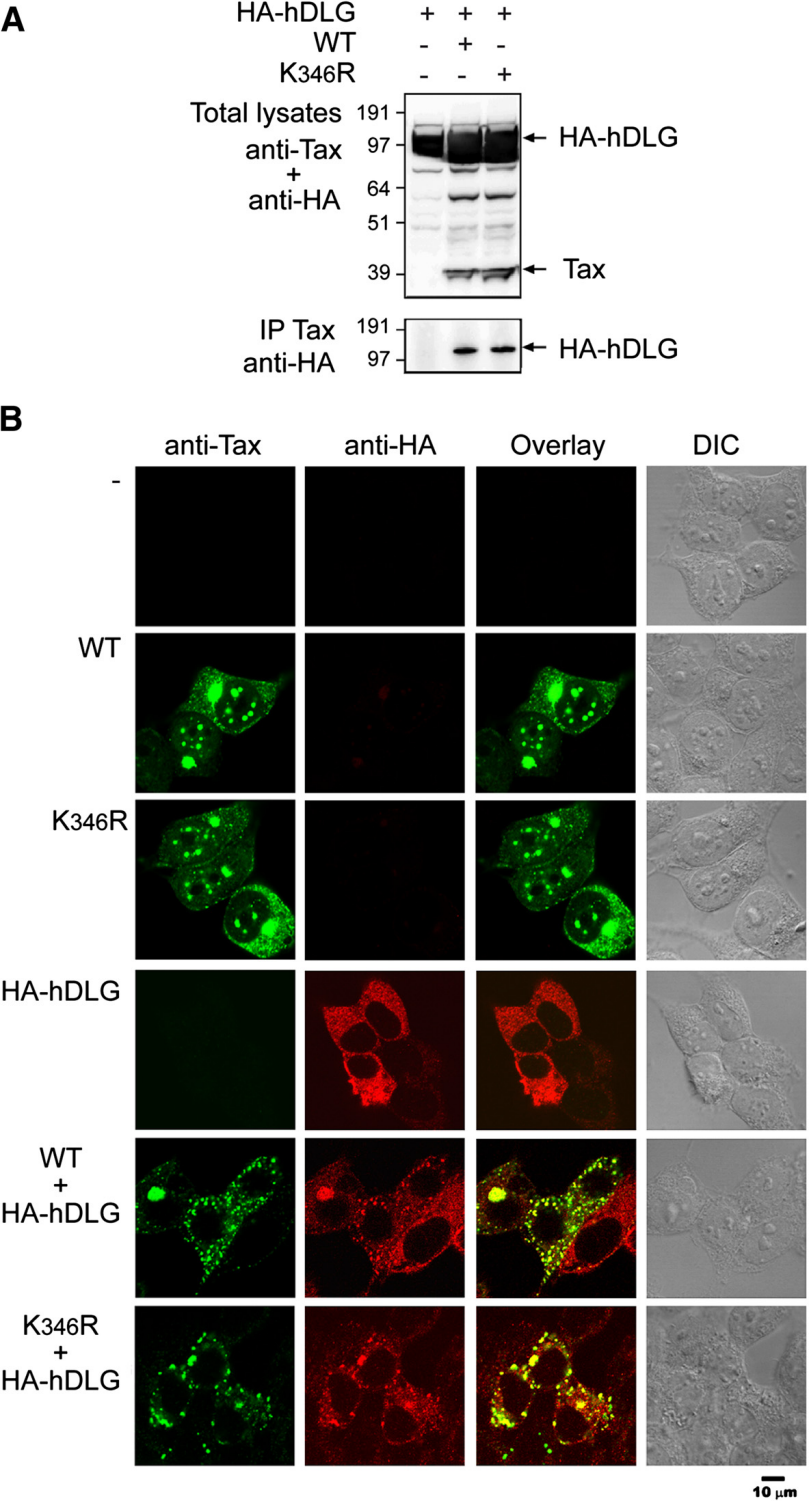
**Acetylation does not prevent interaction of the Tax with PDZ-binding protein hDLG. (A)** 293T cells transfected with vectors expressing HA-hDLG and WT Tax or mutant K_346_R were subjected to anti-Tax immunoprecipitation (IP Tax) and analyzed by Western Blotting with anti-HA antibody. Total lysates were analyzed by Western Blotting with anti-Tax and anti-HA antibodies. **(B)** 293T cells expressing WT Tax or the K_346_R mutant alone or with HA-hDLG were analyzed by immunofluorescence staining and confocal microscopy with anti-Tax IgG2a mAb and anti-HA rabbit polyclonal antibody for the detection of HA-DLG.

Since the acetylation targeted lysine K_346_ lies close to the PBM (E_350_TEV_353_), which controls Tax interaction with hDLG and contributes to Tax transforming activity [33,34], we asked whether Tax acetylation had consequences on the interaction of Tax with hDLG. 293T cells were cotransfected with vectors for expression of either WT Tax or the K_346_R mutant and a vector expressing HA-hDLG. Western Blot analysis of cells lysates immunoprecipitated with anti-Tax revealed that both mutant K_346_R and WT Tax associated with hDLG (Figure 5A**)**. We also tested the intracellular localization of WT Tax or the K_346_R mutant in 293T cells overexpressing HA-hDLG by immunofluorescence staining and confocal microscopy (Figure 5B). Overexpressed HA-hDLG had a rather diffuse distribution in the cytoplasm and coexpression of WT Tax or the K_346_R mutant with HA-hDLG led to the redistribution of both WT or mutant Tax and HA-hDLG to cytoplasmic speckled structures, in which they colocalized. These results support the conclusion that the interaction of the Tax PBM motif with hDLG is not regulated by the acetylation status of Tax.

### Acetylation controls the ability of Tax to stimulate phosphorylation of pRb by CDK4-cyclin D3-p21 complexes

**Figure 6:**
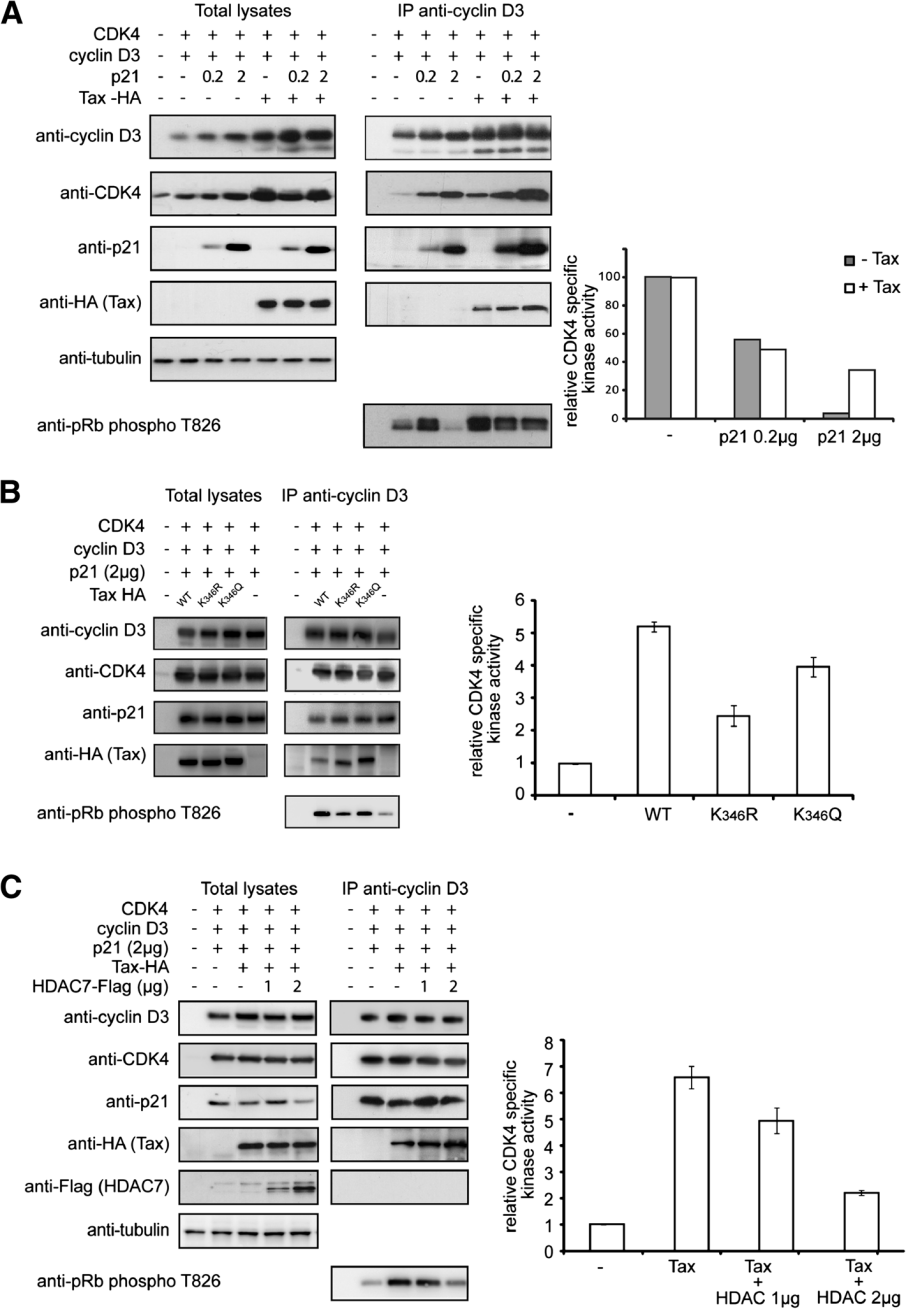
**Acetylation controls the ability of Tax to stimulate phosphorylation of pRb by CDK4-cyclin D3-p21**^**CIP**^**complexes.** CHO cells were transfected with vectors expressing CDK4, cyclin D3 and either 0.2 or 2 μg of vector expressing p21 with or without a vector expressing Tax-HA **(A)**, or 2 μg of vector expressing p21 with vectors expressing WT or mutant K_346_R or K_346_Q Tax-HA **(B)**, or 2 μg of vector expressing p21 and WT Tax-HA and 1 or 2 μg of vector expressing HDAC7-Flag **(C)**. Total lysates were immunoprecipitated with the anti-cyclin D3 mAb and both the total lysates and the immunoprecipitated complexes (IP anti-cyclin D3) were analyzed by Western Blotting using antibodies shown on the left side of the lanes. The immunoprecipitated complexes were also analyzed using an *in vitro* pRb phosphorylation assay. The diagrams represent the relative CDK4 specific activity obtained by quantitation of the intensity of the pRb phosphorylated species on the blots reported to equal amount of CDK4 in the complexes. One representative experiment is presented in panel **A**. Error bars were calculated for two independent experiments in panels **B** and **C**.

Tax-mediated activation of the pRb kinase activity of CDK4/6-cyclin D3-p21^CIP^ complexes contributes to cell transformation by accelerating cell cycle progression at the G1 to S restriction point [26-30]. We thus asked whether Tax acetylation was involved in activation of the pRb kinase activity of these complexes. First, we tested the experimental conditions that led to increased pRb kinase activity of CDK4-cyclin D3 complexes in the presence of Tax (Figure 6A). Intracellular CDK4-D-type cyclin complexes are predominantly associated with CDK inhibitors (CKI) p21 or p27. At low stoichiometric binding ratio, p21 favors formation of active CDK4-cyclin D3 complexes, but higher stoichiometric ratios of p21 binding inhibit CDK4 activity [54-57].

The formation and the kinase activity of the CDK4-cyclin D3-p21 complexes were tested in CHO cells cotransfected with vectors expressing WT Tax-HA, CDK4, cyclin D3 and either low dose (0.2 μg) or high dose (2 μg) of vector expressing p21. The cell extracts were immunoprecipitated with an anti-cyclin D3 mAb and subsequently analyzed by Western Blotting using anti-cyclin D3, anti-CDK4, anti-p21 and anti-HA antibodies to determine the composition of the immunoprecipitated complexes. These complexes were then assayed *in vitro* for phosphorylation of a pRb fragment containing threonine 826, a known target of CDK4. The diagram of Figure 6A represents the relative CDK4 specific kinase activity calculated by estimating the quantity of phosphorylated pRb fragment on the Western Blot reported to equal amount of CDK4 in the complexes.

Expression of p21 in the absence of Tax resulted in stabilization of CDK4-cyclin D3 complexes, in a p21 dose-dependent manner. The specific CDK4 kinase activity of these complexes (grey bars) was reduced by factors 2 and 25 when the complexes were formed in the presence of low or high dose of p21, respectively. These results are in accordance with the reported consequences of p21 dosage on stabilization and inhibition of CDK4-cyclin D3 complexes [56-58]. Stabilization of the CDK4-cyclin D3 complexes in a p21 dose-dependent manner was also observed in the presence of Tax and these complexes included Tax. However, these complexes had a kinase activity (white bars) that was markedly higher than that of the complexes assembled in the absence of Tax, when high dose of p21 was expressed (8.6 fold increase). Expression of Tax in the absence or with a low dose of p21 gave kinase activities similar to that in the absence of Tax. These results indicate that inclusion of Tax in the CDK4-cyclin D3-p21 complexes partly relieves the inhibitory action of p21 on the pRb kinase activity of the complexes. These results are in accordance with previous observations [27,28].

We then tested whether acetylation of Tax had consequences on the formation and kinase activity of the complexes. CHO cells were cotransfected with vectors expressing CDK4, cyclin D3, the high dose (2 μg) of vector expressing p21 and vectors expressing either WT Tax, the non-acetylated K_346_R mutant or the acetylation mimetic K_346_Q mutant (Figure 6B). Both mutants K_346_R and K_346_Q associated with the complexes, but the specific kinase activity of the complexes containing mutant K_346_R was decreased by a factor 2 as compared to complexes containing WT Tax or the acetylation mimetic K_346_Q mutant.

To further analyze the role of Tax acetylation in stimulation of CDK4 kinase activity, we tested whether overexpression of HDAC7, which strongly deacetylates Tax, had consequences on the ability of the CDK4-cyclin D3-p21-Tax complexes to phosphorylate pRb (Figure 6C). Expression of HDAC7 had no consequences on the formation of the CDK4-cyclin D3-p21-Tax complexes, but reduced the ability of these complexes to phosphorylate pRb in a dose-dependent manner. These results indicated that acetylation deficiency did not prevent the association of Tax with CDK4-cyclin D3-p21 complexes, but resulted in a reduced capacity of these complexes to bypass p21 inhibition.

**Figure 7:**
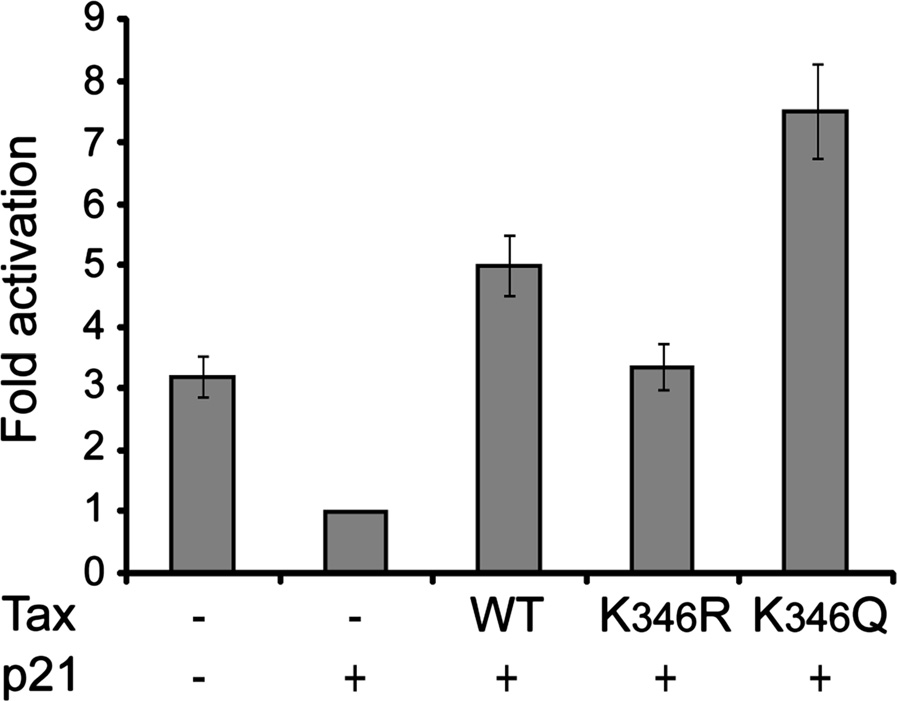
**Acetylation controls Tax-mediated release of free E2F.** CHO cells were transfected with 250 ng of p3xE2F-WT-luc reporter construct and 100 ng of pRL-TK-luc, 50 ng of vectors expressing cyclin D3 and CDK4 alone or in combination with vectors expressing p21 (100 ng), and either WT Tax or the K_346_R or K_346_Q mutants (600 ng). The results are presented as fold increase of luciferase activities. The values are averages of three independent experiments, with standard deviations indicated.

Phosphorylation of pRb results in the release of transcriptionally active free E2F. To further examine the mechanism involved in Tax acetylation-dependent trans-forming activity, we analyzed the luciferase activity of the E2F controlled p3xE2F-luc reporter construct in CHO cells transfected with the plasmids expressing CDK4, cyclin D3 and p21 and either WT Tax or the K_346_R or K_346_Q mutants like reported in Figure 6B. Figure 7 indicates that activation of the E2F controlled promoter was reduced following expression of p21 and that WT Tax expression resulted in the bypass of p21 repression in an acetylation-dependent manner, thus mimicking the effects observed on pRb phosphorylation.

## Discussion

In this work, we have demonstrated that acetylation at lysine K_346_ in the C-terminal domain of Tax plays a critical role in Tax capacity to transform Rat-1 fibroblasts and in rendering the pRb kinase activity of CDK4-cyclin D3-p21-Tax complexes resistant to p21 inhibition. These conclusions are based on the observations that the acetylation deficient K_346_R mutant, but not the acetylation-mimetic K_346_Q mutant, is deficient for both transformation of Rat-1 fibroblasts and activation of the CDK4 complexes stabilized by p21.

Here, we identify two acetylated forms of Tax, including an acetylated and phosphorylated form, that are both present in fibroblasts overexpressing Tax and in HTLV-1 infected T lymphocytes. The fraction of acetylated forms was estimated at about 4 percent of all four 40 kDa Tax species. This low percentage clearly has physiological significance since we demonstrate that loss of Tax acetylation results in markedly reduced transforming activity. It is now well recognized that the biological consequences of modified forms are not proportional to the small fraction of substrate that is modified [59]. It is also worth noting that the acetylated Tax forms are concentrated in the Tax NBs, along with a variety of effectors including the acetyltransferase p300 and the deacetylase HDAC7, which might compete to regulate Tax acetylation status in a dynamic process. Finally, the presence of acetylated Tax in the Tax NBs supports our previous observation using cell fractionation experiments indicating that acetylated Tax is essentially distributed in the nucleus [47]. The contributions of each acetylated form to the transformation phenotype and determination of the phosphorylated residue need further investigations.

In addition to consequences on pRb phosphorylation, our previous studies indicated that acetylated Tax favored activation of a NF-κB controlled promoter, when the reporter construct was integrated in the chromatin [47]. Our results suggest that the transformation phenotype observed in Rat-1 cells does not depend on Tax ability to activate gene expression from a κB-controlled promoter but might depend on constitutive NF-κB activation in Rat-1 cells.

### How does Tax acetylation alter the kinase activity of CDK4?

Mechanistically, acetylation of Tax acts on stimulation of the pRb kinase activity of CDK4-cyclin D3-p21 complexes in CHO cells resulting in increased free E2F available for activation of an E2F-controlled promoter. However, acetylation was dispensable for the association of Tax with the CDK4-cyclin D3-p21 complexes. This concept is supported by the observations that WT Tax as well as mutants K_346_R and K_346_Q were associated with the CDK4-cyclin D3-p21 complexes, and that WT Tax or the acetylation-mimetic K_346_Q mutant were more potent in preventing the inhibitory action of the CDK inhibitor p21 and in increasing transcription from the E2F-controlled promoter than the acetylation deficient K_346_R mutant. In addition, expression of the deacetylase HDAC7, which markedly reduced Tax acetylation, did not affect the association of Tax with the complexes, but prevented stimulation of the pRb kinase activity of the complexes by WT Tax, in a dose-dependent manner.

It will be important to determine how acetylation of Tax acts to prevent the inhibitory action of p21. Binding of p21 through both its cyclin-binding motif (Cy1) and its CDK-binding motif (K) is required for inhibition of the kinase activity of the CDK4-cyclin D complex [60]. Previous studies indicated that the N-terminal 40 amino acids of Tax directly interact with the PSTAIRE N-terminal domain of CDK4 and that Tax also interacts with cyclin D3. Most likely, one of these interactions might affect the binding of p21 through one of its cyclin/CDK interaction motifs. Tax interaction would thus allow the stabilization by p21 of abundant CDK4-cyclin D3 complexes that remain partially active despite high p21 expression levels, leading to enhancement of both phosphorylation of pRb and steady state level of free E2F, with subsequent acceleration of progression from the G1 to the S phase of the cell cycle [27,28]. In addition, Tax stimulates expression of p21 at the transcriptional level in fibroblasts overexpressing Tax and in HTLV-1 infected T lymphocytes [61,62], and induces phosphorylation of cyclin D3 [26]. Importantly, our results indicate that lack of acetylation does not affect the ability of Tax K_346_R to associate with CDK4-cyclin D3-p21 complexes. The K_346_ acetylated C-terminal domain of Tax might recruit a kinase involved in phosphorylation of critical residues of the CDK4-cyclin D3-p21 complex such as threonine 172 in the CDK4 activation loop [58]. Alternatively, since an extensive number of kinases regulate CKIs to control their function, localization and stability [63], one could speculate that Tax recruits a kinase involved in the control of p21 inhibitory function.

Previous studies indicated that, in T lymphocytes cell lines and HTLV-1 infected lymphocytes, but not in fibroblasts, Tax activates cyclin D2 and CDK6 promoters via activation of the NF-κB pathway resulting in cell cycle progression [64]. In addition, stable p21-cyclin D2-CDK4 complexes active in phosphorylation of pRb were detected in HTLV-1 infected T cells [65]. Further studies will be important to determine whether Tax acetylation also controls cell cycle progression in T-cells and whether the activity of different complexes such as p21-cyclin D2-CDK6 are modulated by Tax acetylation in T cells.

### How does Tax C-terminal region function in cellular transformation?

The acetylation targeted lysine K_346_ is part of the C-terminal domain of Tax that otherwise contains two critical functions participating in Tax transforming potential. These include the four C-terminal amino acids which form a PBM motif for interaction with the hDLG and hScrib and MAGI-1 tumor suppressors [11,31,33,34], and a region involved in micronuclei formation [48,66]. Here we show that the lack of acetylation of the K_346_R mutant does not affect its ability to interact with hDLG or to be redistributed by hDLG into cytoplasmic speckles. We conclude that activation of the kinase activity of CDK4-cyclin D3-p21 complexes by acetylated Tax and interaction with hDLG are distinct functions that both participate in Tax transforming activity, in a necessary, but not sufficient manner.

Comparison of the transforming activities of the Tax proteins of HTLV-1 (Tax-1) and HTLV-2 (Tax-2), which markedly differ in pathogenicity, might reveal information about the role of the C-terminal domain in Tax-induced transformation. The weakly transforming Tax-2 protein shares a high degree of amino acid similarity with Tax-1, but the two proteins have divergent C-terminal domains. In addition, Tax-2 does not include a functional PBM and does not induce micronuclei formation [67]. Furthermore, the chimera formed by fusion of the C-terminal domain of Tax-1 to the C-terminus of Tax-2 displays increased capacity to promote proliferation of human PBMCs as compared to Tax-2 [34,68]. Studies are underway to determine whether the acetylation status of Tax-2 concurs with the absence of a PBM to determine its reduced transforming activity.

### How do Tax NBs function in cellular transformation?

Tax NBs control two central functions in Tax transforming activity. These include activation of gene expression via the NF-κB pathway [39,40], and sequestration of effectors involved in DNA repair [24,25]. Our finding showing that the acetylated forms of Tax and the enzymes that modulate Tax acetylation are concentrated in Tax NBs, incriminates these structures in both Tax acetylation and transforming activity. This conclusion is also supported by our previous observation that mutants deficient for sumoylation and subsequent assembly of the Tax NBs have a markedly reduced acetylation status [47]. These results support the idea that transformation by Tax is the consequence of a cascade linking Tax sumoylation-dependent acetylation and activation of cyclin-dependent kinase CDK4 for cell cycle progression.

## Conclusions

### Is deacetylase therapy appropriate for ATL?

Our work questions whether treatments of ATL patients with drug regimens that include inhibitors of deacetylases are appropriate since such treatments could theoretically increase Tax acetylation [69,70]. It will be important to determine if Tax acetylation promotes transformation of HTLV-1 infected T lymphocytes and if HDAC inhibitors stimulates Tax acetylation in T-cells. Determination of the actual enzymes that regulate Tax acetylation in HTLV-1 infected patient samples would provide new therapeutic targets for the treatment of HTLV-1 infected patients who present a risk of developing ATL.

## Methods

### Cell culture and transfection

293T, 293GP2, HeLa and Rat-1 cells were maintained in Dulbecco’s modified Eagle’s medium (DMEM), and CHO cells were maintained in F12 HAM medium, which were supplemented with 2 mM L-Glutamine, 10% fetal calf serum (FCS), 1% penicillin-streptomycin and 1 mM sodium pyruvate (Life technologies, Paisley, UK). Cell lines were transfected using the Transit LT-1 reagent (Mirus Bio LLC, Madison, USA) according to the manufacturer’s instructions. The HTLV-1 infected T lymphocyte cell lines C8166 and MT4 were maintained in RPMI medium with Glutamax-I (Life technologies, Paisley, UK) supplemented with 10% FCS. Expression of Tax by JPX9, a derivative of Jurkat cells containing an inducible *tax* gene under the control of the metallothionein promoter, was induced by supplementing 30 μM CdCl_2_ to the RPMI medium for 16 h [71]. Trichostatin A (TSA) was purchased from Sigma-Aldrich (Belgium).

### Expression vectors

The vectors for expression of WT or K_346_R Tax mutant and p300-HA were previously described [47], as well as vectors for expression of CDK4 and cyclin D3 [58]. Mutant K_346_Q was generated by PCR based site-directed mutagenesis. Vector for expression of HA-hDLG was provided by L. Banks [72], HDAC7-Flag was from F. Dequiedt [73], and p21^CIP^ (referred to as p21) was from L. Hengst (Innsbruck). The luciferase reporter constructs κB-luc (Promega), HTLV-1-LTR-luc [39], and p3xE2F-luc [74] were used in luciferase assays.

### Transformation of Rat-1 fibroblasts in soft agar

For lentiviral vector construction, WT, K_346_R and K_346_Q Tax coding sequences were inserted into the pCSEF-IRES-bsd lentiviral vector kindly provided by M. Fujii [11]. This generated the pCSEF-IRES-bsd control, pCSEF-Tax WT, pCSEF-Tax K_346_R and pCSEF-Tax K_346_Q lentiviral vectors, which were transfected in 2 × 10^6^ 293GP2 packaging cells together with pCAG-HIVgp and pCMV-VSV-G-RSV-Rev to produce viral particles. Production of lentiviral vectors expressing Tax, stable transduction of Rat-1 cells and transformation assays have been described [20].

### Antibodies

The antibody recognizing the K_346_ acetylated form of Tax was obtained by immunizing rabbits with a synthetic peptide (H_2_N-CEPPSEK(Ac)HFRET-CONH_2_). Specific antibodies were enriched by selection against both the acetylated and the non-acetylated peptides (Eurogentec, Seraing, Belgium). The specificity of the purified antibody was tested by ELISA (Additional file 3). The mouse IgG2a anti-Tax-1 mAb from hybridoma 168-A51 was obtained from the AIDS Research and Reagent Program, National Institutes of Health (Bethesda, MD, USA). Anti-p300 (RW-128) was from Upstate (Massachusetts, USA). Anti-CDK4 (H-22), anti-cyclin D3 (DSC-28), anti-RelA (C-20) and anti-p21 (C-19) antibodies were from Santa Cruz Biotechnologies (California, USA). Anti-actin, anti-Flag (M2) and the biotinylated anti-HA antibodies were from Sigma (St. Louis, USA). Anti-phospho T826 pRb antibody was from Abcam (Cambridge, UK). The secondary antibodies used for immunofluorescence staining were Dylight 488-conjugated goat anti-mouse IgG2a, Dylight 549- or 488-conjugated goat anti-rabbit IgG and Dylight 649-conjugated goat anti-mouse IgG1 from Jackson ImmunoResearch (Pennsylvania, USA).

### Luciferase activities

Tax-mediated transactivation of genes expression via the NF-κB and ATF/CREB pathway was evaluated by dual luciferase assays using of the transfected κB-luc and HTLV-1-LTR-luc reporter constructs respectively. Rat-1 cells (1 × 10^5^) transducted with either the control lentivirus or with the lentivirus expressing wild type Tax were transfected into 12-well plates with 500 ng of pRL-TK-luc (Promega), used for monitoring transfection efficiency, and either 500 ng of κB-luc or 500 ng of HTLV-1-LTR-luc reporter plasmids. 24 h after transfection, cells were lysed and subjected to luciferase assay with the dual luciferase reporter assay system (Promega) and a TD-20/20 luminometer (Turner Designs) according to protocol of the manufacturer. For E2F luciferase assays, CHO cells were transfected with 250 ng of p3xE2F-WT-luc and 100 ng of pRL-TK-luc reporter plasmids, and vectors expressing cyclin D3 and CDK4 (50 ng each). The effects of p21 CDK inhibitor and WT Tax or K_346_R or K_346_Q mutants on activation of the E2F-controlled promoter were assessed by cotransfection of p21 (100 ng) and Tax (600 ng) expression vectors.

### Two-dimensional (2D) gel electrophoresis

Cells were lysed in lysis buffer containing 7 M urea, 2 M thiourea, 40 mM DTT, 4% (w/v) CHAPS and deacetylase, protease and phosphatase inhibitors (0.5 μM TSA and 5 mM NAA, complete EDTA-free and Phosphostop from Roche Diagnostics GmbH, Mannheim, Germany). Immunoglobulins (0.4 μg) were used as internal pH markers and added to the sample before loading. Proteins were separated by isoelectric focusing using the Ettan IPGphor3 apparatus from GE Healthcare (Buckinghamshire, UK) and active in-gel rehydratation of pH5-8 IPG strips as described in manufacturer’s protocol (BioRad, California, USA). After isoelectric focusing, the IPG strips were loaded onto 4-12% Bis-Tris NuPAGE gel (Life technologies, Paisley, UK) for separation according to molecular mass. Methods for second dimension electrophoresis and Western Blotting have been described [47].

### Co-immunoprecipitation and *in vitro* pRb phosphorylation assay

Co-immunoprecipitation and *in vitro* pRb phosphorylation assay were performed according to [58]. Briefly, cells were lysed in buffer containing 150 mM NaCl, 50 mM Tris–HCl pH 7.5, 0.5% Nonidet P-40, 50 mM NaF, 1 mM sodium orthovanadate, 1 mM β-glycerophosphate, 10 mM DTT, protease inhibitors (complete EDTA-free, Roche Diagnostics GmbH, Mannheim, Germany), and 10% glycerol. The homogenized cellular lysates were cleared and incubated at 4°C for 3 h with protein A sepharose 4 fast flow that had been preincubated overnight with 2 μg of the antibody used for immunoprecipitation. Immunoprecipitated complexes were resuspended in kinase reaction buffer (50 mM HEPES, pH 7.5, 10 mM KCl, 10 mM MgCl_2,_ 2.5 mM EGTA, 1 mM DTT) containing 2 mM ATP, 0.5 μg of a pRb fragment (amino acids 773–928) (Sigma, St. Louis, USA), 10 mM β-glycerophosphate, 0.1 mM orthovanadate, 1 mM NaF, protease inhibitors (complete EDTA-free, Roche Diagnostics GmbH, Mannheim, Germany), and incubated for 30 min at 30°C. Immunoprecipitated complexes and kinase assays were separated on 4-12% Bis-Tris NuPAGE gel (Life technologies, Paisley, UK) and analyzed by Western Blotting. The detection was performed using the ECL Advance Western Blotting detection kit (GE Healthcare, Buckinghamshire, UK) and quantitation of chemiluminescent signals was performed with the Chemi-Smart 5000 apparatus and Bio-1D software (Vilber Lourmat, Marne-la-Vallée, France).

### Immunofluorescence and confocal microscopy

Cells were grown on glass coverslips and fixed with immunohistofix (Gentaur, Kampenhout, Belgium) for 10 min at room temperature (RT) followed by methanol for 6 min at −20°C and further processed as described previously [39].

## Competing interests

The authors declare that they have no competing interest.

## Authors’ contributions

JL, CS, MB, ASR and KC made the experiments. JL wrote the manuscript. LW, PPR and FB supervised the work and corrected the manuscript. All authors read and approved the final manuscript.

## Supplementary Material

Additional file 1 **Treatment of Tax expressing cells with HDAC inhibitor TSA increases Tax acetylation.** 293T cells expressing Tax were treated for 18 h with TSA at the indicated concentrations and analyzed by Western Blotting using anti-Tax, anti-AcK_346_Tax and anti-actin antibodies. The numbers under the blots represent relative quantities of each Tax species normalized to equal amount of actin. Click here for file

Additional file 2 **Detection of phosphorylated forms of Tax.** 293T cells were transfected with a vector expressing WT Tax and metabolically labeled with ^32^P orthophosphate. The cell extracts were separated by two-dimensional gel electrophoresis and analyzed by Western Blotting with the anti-Tax mAb and autoradiography. Quantitation of the NM (non-modified), Ac (acetylated), P (phosphorylated) and Ac/P (acetylated and phosphorylated) species on the anti-Tax immunoblot was done using Image J software. Click here for file

Additional file 3 **Specificity of anti-AcK**_**346**_**Tax.** ELISA was performed by coating 96-well plates with 100 ng of either the non acetylated peptide or the acetylated peptide that was used for rabbit immunization. Serial dilutions of the anti-AcK_346_Tax antibody (4.6 mg/ml) followed by anti-rabbit-HRP were incubated for 2 hours. The substrate was incubated for 30 min and the reaction was stopped with 4 M H_2_SO_4,_ followed by optical density measurements at 492 nm. Click here for file
