# Supplementary figures and images for: Acetylation at lysine 346 controls the transforming activity of the HTLV-1 Tax oncoprotein in the Rat-1 fibroblast model
Source: Retrovirology. 2013 Jul 23;10:75. doi: 10.1186/1742-4690-10-75 (PMC3734113; doi:10.1186/1742-4690-10-75)

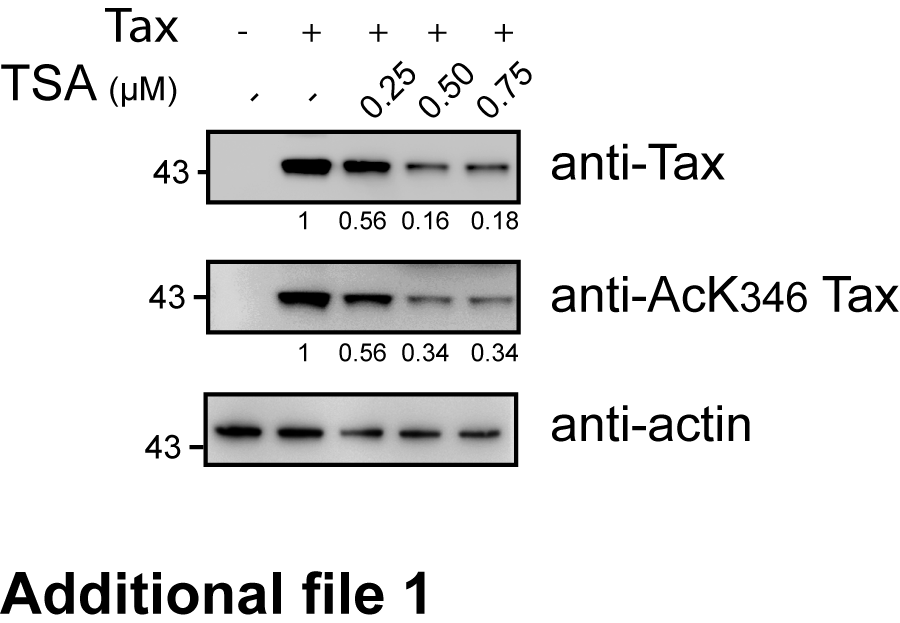

Supplement: Additional file 1 — Treatment of Tax expressing cells with HDAC inhibitor TSA increases Tax acetylation. 293T cells expressing Tax were treated for 18 h with TSA at the indicated concentrations and analyzed by Western Blotting using anti-Tax, anti-AcK346Tax and anti-actin antibodies. The numbers under the blots represent relative quantities of each Tax species normalized to equal amount of actin. [file 1742-4690-10-75-S1.tiff]

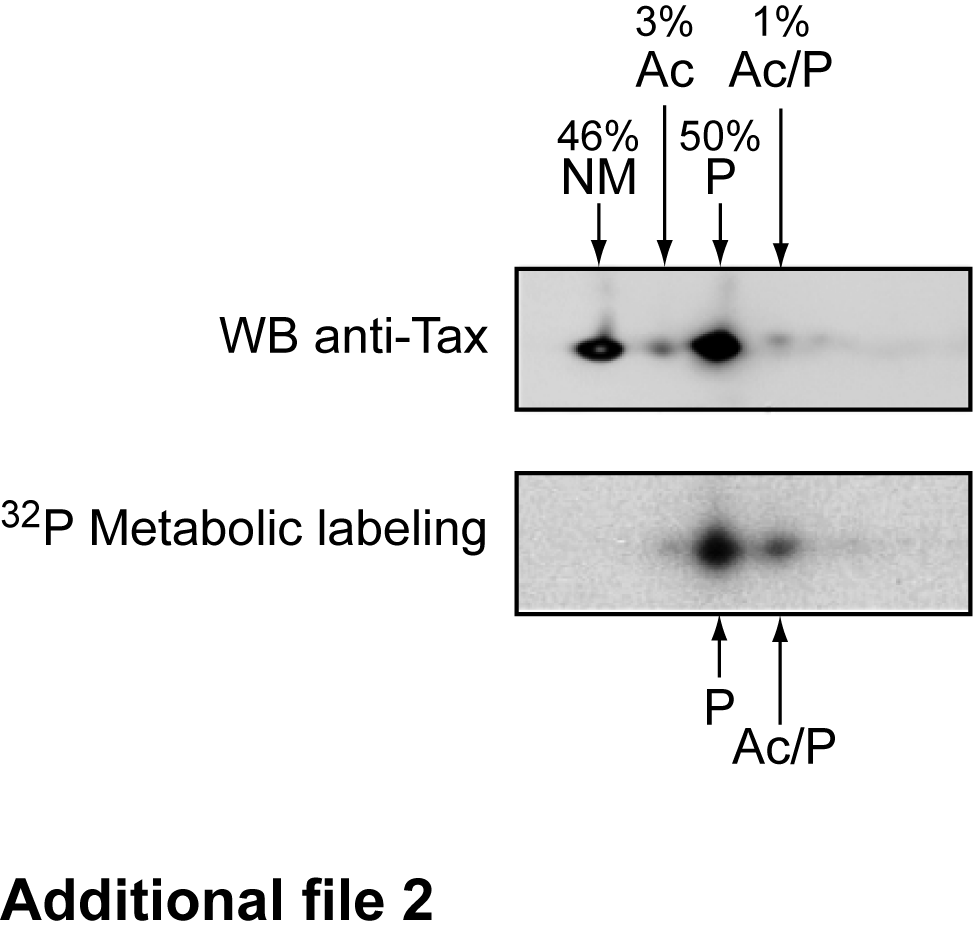

Supplement: Additional file 2 — Detection of phosphorylated forms of Tax. 293T cells were transfected with a vector expressing WT Tax and metabolically labeled with 32P orthophosphate. The cell extracts were separated by two-dimensional gel electrophoresis and analyzed by Western Blotting with the anti-Tax mAb and autoradiography. Quantitation of the NM (non-modified), Ac (acetylated), P (phosphorylated) and Ac/P (acetylated and phosphorylated) species on the anti-Tax immunoblot was done using Image J software. [file 1742-4690-10-75-S2.tiff]

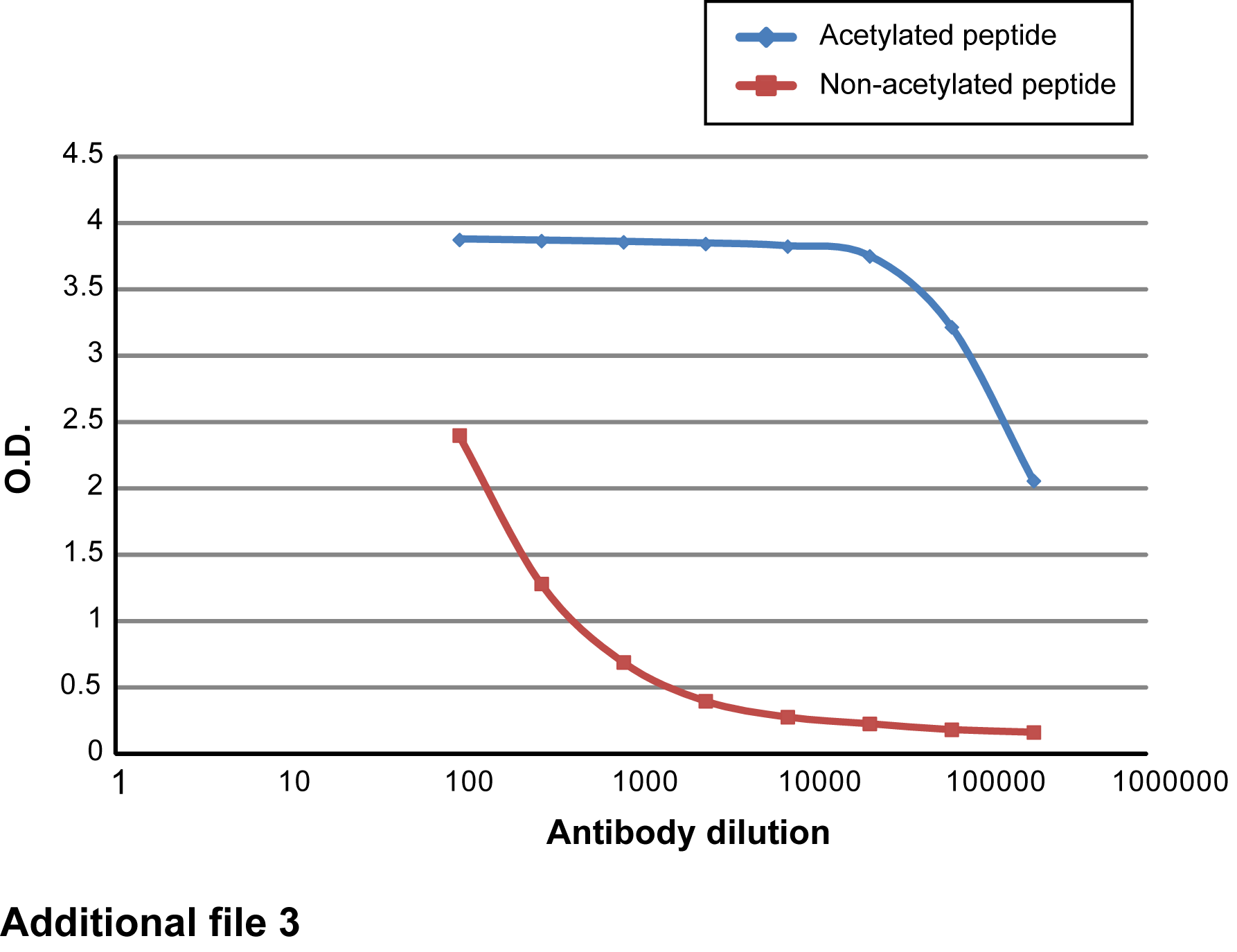

Supplement: Additional file 3 — Specificity of anti-AcK346Tax. ELISA was performed by coating 96-well plates with 100 ng of either the non acetylated peptide or the acetylated peptide that was used for rabbit immunization. Serial dilutions of the anti-AcK346Tax antibody (4.6 mg/ml) followed by anti-rabbit-HRP were incubated for 2 hours. The substrate was incubated for 30 min and the reaction was stopped with 4 M H2SO4, followed by optical density measurements at 492 nm. [file 1742-4690-10-75-S3.tiff]
